# Four cycles of R-CHOP followed by two applications of rituximab based on negative interim PET/CT: an analysis of a prospective trial

**DOI:** 10.1186/s12885-022-09486-4

**Published:** 2022-04-13

**Authors:** Jia Jin, Dongmei Ji, Zuguang Xia, Kai Xue, Qunling Zhang, Yizhen Liu, Junning Cao, Xiaonan Hong, Juan J. Gu, Ye Guo, Fangfang Lv

**Affiliations:** 1 Department of Medical Oncology, Fudan University Shanghai Cancer Center, 270 Dong’an Road, Shanghai, 200032 China; 2 Department of Oncology, Shanghai Medical College, Fudan University, Shanghai, 200032 China; 3 Department of Hematology, Shanghai Jiao Tong University School of Medicine, Affiliated Ruijin Hospital, Shanghai, 200025 China; 4 Oncology Department, Northern Jiangsu People;s Hospital; Cancer Institute affiliated to Northern Jiangsu People’s Hospital; Medical College, Yangzhou University, Yangzhou, 255000 Jiangsu Province China; 5 Department of Oncology, Shanghai East Hospital, Tongji University, Shanghai, 200120 China

**Keywords:** Diffuse large B-cell lymphoma, Limited stage, Positron emission tomography, Prognosis

## Abstract

**Background:**

R-CHOP with or without radiotherapy is the standard treatment for limited-stage diffuse large B-cell lymphoma (DLBCL). To prevent overtreatment, we assessed whether four cycles of CHOP plus six applications of rituximab was adequate with negative interim PET/CT and the role of consolidation radiotherapy specifically for patients with Waldeyer’s ring DLBCL. One hundred and twenty-nine patients with limited-stage DLBCL were enrolled in this open-label, nonrandomized, single-arm, phase 2 clinical trial (NCT01804127).

**Methods:**

All patients were initially treated with 4 cycles of R-CHOP and underwent interim PET/CT. Patients with negative PET/CT (Deauville scores 1–2) received 2 additional cycles of rituximab monotherapy, unless they had any risk factors (primary mediastinal large B-cell lymphoma, extranodal primary or bulky disease). Otherwise, patients received another 2 cycles of R-CHOP. Patients with partial response on interim PET/CT received another 4 cycles of R-CHOP. No radiotherapy was conducted in Waldeyer’s ring DLBCL patients with negative PET/CT. The primary endpoint was 3-year progression-free survival (PFS). Overall survival (OS) in this study was compared with those from a historical study (NCT 00854568159).

**Results:**

One hundred fifteen interim PET/CT scans (89.1%) were negative after 4 cycles of R-CHOP. An elevated lactate dehydrogenase level was significantly associated with positive interim PET/CT (*P* < 0.05). A trend of inferior outcome was observed in patients with positive interim PET/CT in terms of 3-year PFS (78.6% vs. 91.9%, *P* = 0.24) and 3-year OS (85.7% vs. 95.6%, *P* = 0.16). There were no PFS or OS differences found between patients treated with 4R-CHOP+2R and those treated with 6R-CHOP from a historical control study. Patients with Waldeyer’s ring DLBCL and negative interim PET/CT achieved a 3-year PFS of 87.2% and a 3-year OS of 89.7%.

**Conclusions:**

Our results suggested that for interim PET/CT-negative patients without risk factors, the extra 2 cycles of CHOP might be omitted, and radiotherapy might also be omitted in patients with Waldeyer’s ring DLBCL without compromising the efficacy. These results need to be confirmed in a randomized study.

**Trial registration:**

clinicaltrials.gov, NCT 01804127. Date of first registration: 05/03/2013.

## Background

Diffuse large B-cell lymphoma (DLBCL) is the most common subtype of malignant lymphoma and accounts for approximately 40% of non-Hodgkin lymphoma cases worldwide [1, 2].

The CHOP (cyclophosphamide, doxorubicin, vincristine and prednisone) regimen has been the standard chemotherapy regimen for the treatment of DLBCL [3]. In recent years, the use of rituximab, whether for elderly patients (GELA LNH-98.5 and RICOVER-60 studies) or young patients (MinT study), on the basis of the CHOP regimen (R-CHOP) has significantly improved the response rate and survival [4–8]. Currently, R-CHOP with or without radiotherapy is the standard treatment for limited-stage DLBCL, and approximately 60–70% of DLBCL patients can be cured using immune-chemotherapy [9].

To achieve a better response, there have been many efforts to intensify the treatment of R-CHOP, including shortening the interval of therapy and increasing the number of cycles in the early stages. However, two studies in France and the United Kingdom suggested that shortening the interval (R-CHOP14 regimen) failed to have additional benefit based on the conventional R-CHOP21 regimen [10].

Previous studies on elderly patients usually used 8 cycles of rituximab, but the number of cycles of chemotherapy is inconsistent from study to study. There were 8 cycles in the GELA LNH-98.5 study and 6 cycles in the RICOVER-60 study. The latter found that with the combination of rituximab, 8 cycles of chemotherapy even tended to decrease survival [4–6]. For young patients (MinT study), 6 cycles of R-CHOP were applied [6, 7]. Since 2011, the NCCN guidelines have revised the treatment recommendations for stage III/IV DLBCL, from the previous 6–8 cycles of the R-CHOP regimen to 6 cycles of the R-CHOP regimen.

Since then, an increasing number of studies have focused on de-escalation therapy without compromising efficacy. To prevent overtreatment, the DSHNHL FLYER study enrolled young, very low-risk stage I/II B-cell non-Hodgkin lymphoma patients. They found that 4 cycles of CHOP plus 6 applications of rituximab was noninferior to 6 cycles of R-CHOP [11].

^18^F-fluoro-2-deoxy-D-glucose (^18^F-FDG) positron emission tomography/computed tomography (PET/CT) is a new type of nuclear medicine assessment that is currently recommended for the response assessment of Hodgkin and non-Hodgkin lymphomas [11]. In recent years, many studies have been conducted focused on the prognostic value of interim PET/CT in lymphoma. Studies have shown that interim PET/CT scans after 2–4 cycles of treatment are an independent prognostic factor in DLBCL patients. The treatment outcomes of interim PET/CT-negative patients are significantly better than those of interim PET/CT-positive patients [12–16]. In the two studies in which the R-CHOP regimen was used, the 3-year OS rates of interim PET/CT-negative patients reached 93.8 and 88%, suggesting that these patients had a good prognosis [15, 16]. Therefore, by interim PET/CT assessment, it is possible not only to guide treatment escalation in poor responders to improve remission rates but also to screen out patients with a good prognosis to prevent overtreatment.

Currently, an interim PET/CT-guided therapeutic approach is being actively explored in limited-stage DLBCL. However, few published data are available at present. The Canadian trial requested an interim PET/CT scan after 3 cycles of R-CHOP. For those who obtained negative results, just one more cycle was added [17]. Otherwise, involved-field radiotherapy was carried out. After 3 cycles of R-CHOP, more than 75 % of patients were interim PET/CT-negative. These patients had very good survival rates, with a 3-year time to disease progression of 92% and a 3-year overall survival (OS) of 96%. The preliminary results suggest that interim PET/CT may be a useful tool to guide de-escalation treatment in good responders.

Despite its high cost, PET/CT has been gradually recognized as an essential baseline and end-of-treatment assessment in China since the 2010s. Emerging studies on interim PET/CT are mostly conducted in developed countries. Since the interval of R-CHOP therapy had been fixed (R-CHOP21 regimen), to keep up with global standardization and individualized treatment, we are committed to carrying out de-escalation treatment trials to explore the number of cycles of chemotherapy.

The standard treatment of Waldeyer’s ring DLBCL with limited stage remains controversial. Li et al. [18] conducted a retrospective study in our centre and found that consolidation radiotherapy did not improve survival in patients with limited Waldeyer’s ring DLBCL after complete remission with R-CHOP.

Therefore, we conducted an open-label, nonrandomized, single-arm, phase 2 clinical trial using response-adapted therapy for limited-stage DLBCL based on interim PET/CT. We assessed whether four cycles of CHOP plus six applications of rituximab was adequate with negative interim PET/CT and the role of consolidation radiotherapy, especially for patients with Waldeyer’s ring DLBCL, to prevent overtreatment.

## Methods

### Inclusion and exclusion criteria

#### Patients

Patients aged between 18 and 80 years with histologically confirmed DLBCL were eligible for inclusion in the study. Patients with limited stage were required to have an Eastern Cooperative Oncology Group (ECOG) performance status (PS) score of no more than 2 and evidence of adequate organ function. Prior treatment was not allowed. Patients with primary or secondary central nervous system involvement, a known history of other malignant tumours, a known history of HIV or HBV-DNA copies higher than the test value were excluded.

#### Treatment and response evaluation

All patients underwent baseline PET/CT within two weeks before the commencement of therapy and had positive and measurable lesions on PET/CT. Then, they were initially treated with 4 cycles of R-CHOP (rituximab 375 mg/m2 d1; cyclophosphamide 750 mg/m2 d2; doxorubicin 50 mg/m2 d2; vincristine 1.4 mg/m2 [maximum 2 mg] d2; prednisone 100 mg orally daily d2–6). R-CHOP was administered every 3 weeks.

An interim PET/CT scan was performed after 4 cycles of R-CHOP on Cycle 4 Day 18 to Day 20 if patients had not progressed after 2 cycles. The Lugano criteria (Cheson 2014) [19] were used for the evaluation of the therapy response. Patients with negative PET/CT (Deauville scores 1–2) received 2 cycles of rituximab monotherapy unless they had any risk factors (primary mediastinal large B-cell lymphoma, extranodal primary or bulky disease). Patients with the abovementioned risk factors received another 2 cycles of R-CHOP as routine practice. Patients with positive PET/CT but achieving partial response (PR) received another 4 cycles of R-CHOP and repeated PET/CT scans at the end of treatment. To prevent undertreatment, patients with a Deauville score of 3 were considered interim PET/CT-positive, and their treatments were the same as those for patients with PR. Patients with stable disease or progressive disease were managed by salvage chemotherapy. After the completion of therapy, the patients were followed up every 3 months for the first 2 years and then every 6 months for 3 years.

#### Statistical analysis

The primary endpoint was 3-year progression-free survival (PFS), and the secondary endpoints included 3-year OS and objective response rate (ORR). PFS was defined as the interval between the initiation of R-CHOP treatment and disease progression or death from any cause. OS was calculated from the date of the initiation of R-CHOP treatment to the date of death from any cause or the last follow-up.

The PFS and OS of negative interim PET/CT patients in this study (treated with 4R-CHOP+2R) were compared with those from a historic group of patients (*n* = 128) treated with 6 cycles of R-CHOP in our centre between March 2009 and December 2012 (NCT 00854568159) [20].

Categorical variables are expressed as frequencies. The chi-square test was applied to detect differences between groups. PFS and OS were calculated using Kaplan-Meier analysis, with differences between groups compared using the log-rank test, and a difference with *P* < 0.05 was considered significant. Univariate and multivariate analyses for survival were performed by the Cox regression model. All statistical analyses were performed using SPSS 17.0 software (SPSS Inc., Chicago, IL, USA).

#### Ethical approval

The study was approved by the institutional review board of Fudan University Shanghai Cancer Center. The trial was registered with ClinicalTrials.gov (number: NCT 01804127, date of first registration: 05/03/2013). All patients provided written informed consent.

## Results

### Patient characteristics

**Fig. 1:**
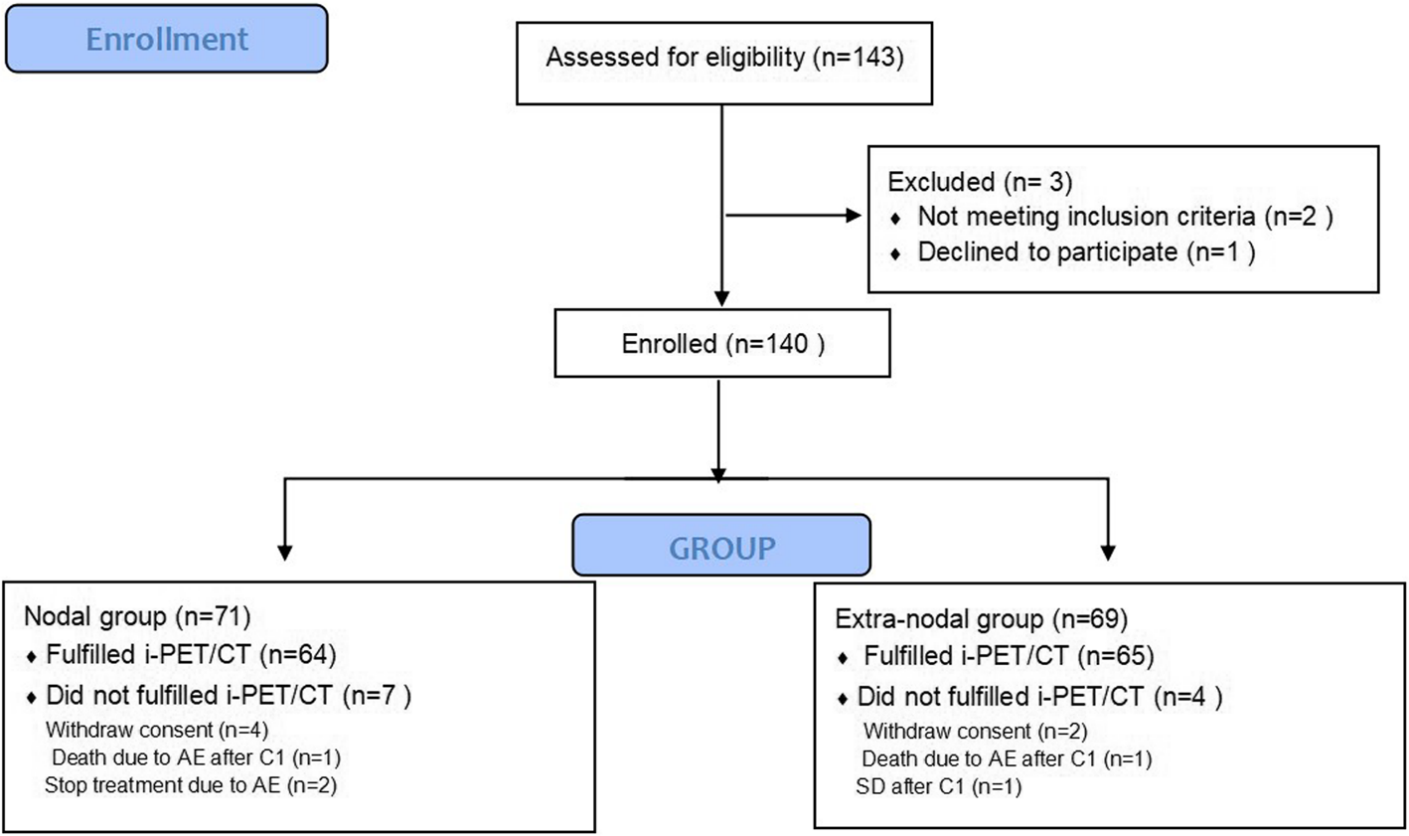
Patient distribution. CONSORT diagram of the allocation and disposition of patients with limited-stage diffuse large B-cell lymphoma treated in this open-label, nonrandomized, phase 2 clinical trial (NCT 01804127)

From December 2012 to September 2015, a total of 143 patients were enrolled. Among these patients, 129 with baseline and interim PET/CT scans were analysed to evaluate the efficacy at Fudan University Shanghai Cancer Center, Shanghai, China (Fig. 1). All patients were followed until death or up to December 2020. The median follow-up time was 52.5 months (range, 7.0–95.6 months).

The patients’ demographic and clinical characteristics are presented in Table 1.

**Table 1:** Patients’ Characteristics According to Interim PET/CT Status

Characteristic	interim PET/CT Negative, *n* = 115		interim PET/CT Positive, *n* = 14	Total, *N* = 129 N 51334	χ^2^	P
	4R-CHOP+2R,*n* = 63	6R-CHOP, *n* = 52				
Age, year						
Median (range)	47.9 (18–74)	53 (24–77)	48.5 (20–70)	50 (18–77)		
≤60y	51 (81)	42 (80.8)	10 (71.4)	103 (79.8)	0.23	0.63
> 60y	12 (19)	10 (19.2)	4 (28.6)	26 (20.2)		
Gender						
Male	36 (57.1)	26 (50)	10 (71.4)	72 (55.8)	0.92	0.33
Female	27 (42.9)	26 (50)	4 (28.6)	57 (44.2)		
Extranodal involvement						
No	63 (100)	1 (1.9)	6 (42.9)	70 (54.3)	0.39	0.53
Yes	0	51 (98.1)	8 (57.1)	59 (45.7)		
Ann Arbor Stage						
I	27 (42.9)	25 (48.1)	4 (28.6)	56 (43.4)	0.81	0.37
II	36 (57.1)	27 (51.9)	10 (71.4)	73 (56.6)		
ECOG PS						
0	40 (63.5)	27 (51.9)	6 (42.9)	73 (56.6)	0.65	0.41
1	23 (36.5)	25 (48.1)	8 (57.1)	56 (43.4)		
B symptoms						
No	61 (96.8)	46 (88.5)	13 (92.9)	120 (93)	0.28	0.59
Yes	2 (3.2)	6 (11.5)	1 (7.1)	9 (7)		
LDH level						
Normal	60 (95.2)	51 (98.1)	11 (78.6)	122 (94.6)	4.72	0.03*
Elevated	3 (4.8)	1(1.9)	3 (21.4)	7 (5.4)		
IPI score						
0–1	61 (96.8)	51 (98.1)	14 (100)	116	0.11	0.74
2	2(3.2)	1 (1.9)	0 (0)	3 (2.3)		

Values shown are n (%) or median (range)

Abbreviations: *PS* Performance Status, *LDH* Lactate Dehydrogenase, *IPI* International Prognostic Index

Chi-square test was applied to detect differences of characteristics between interim PET/CT positive (n = 14) and negative ones (n = 115). * *P* < 0.05

A total of 57 women (44.2%) and 72 men (55.8%) were included. The median age was 50 years (range, 18–77 years). In the entire cohort, 73 (56.6%) of 129 patients had stage II disease, and 9 (7%) of 129 patients exhibited B symptoms. With regard to risk factors (primary mediastinal large B-cell lymphoma, extranodal primary or bulky disease), 59 (45.7%) of 129 patients had extranodal primary lesions, and 1 (0.8%) of 129 patients had a bulk > 7.5 cm on CT. Based on the International Prognostic Index (IPI) score, 126 (97.7%) patients had a low risk of relapse (Table 1).

### Correlation of interim PET/CT results with survival

Based on the Deauville criteria, 115 PET/CT scans (89.1%) were reported as negative (Deauville scores 1–2). According to the protocol, 63 (54.8%) of 115 patients with negative interim PET/CT scans and no risk factors were treated with 4R-CHOP+2R. Fifty-two (45.2%) of 115 patients with negative PET/CT scans were treated with 6R-CHOP (51 patients with extranodal involvement and 1 patient with bulky disease).

Among 14 patients with positive PET/CT, 13 had PR, and 1 had stable disease. The ORR was 99.2%. With a median follow-up time of 52.5 months, the 3-year PFS rate was 90.5%, and the 3-year OS rate was 94.5% for the entire cohort (*n* = 129).

**Fig. 2:**
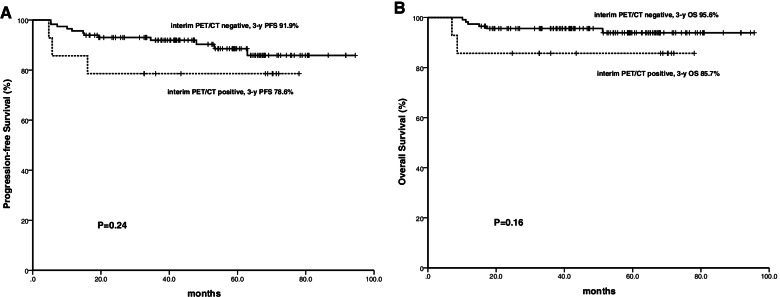
Progression-free survival (**A**) and overall survival (**B**) in 129 patients based on interim PET/CT scan results

A trend of inferior outcome was observed in patients with positive interim PET/CT in terms of the 3-year PFS (78.6% vs. 91.9%, *P* = 0.24) and 3-year OS (85.7% vs. 95.6%, *P* = 0.16). However, the differences were not statistically significant (Fig. 2).

### Correlation of interim PET/CT status with demographic and clinical characteristics

The chi-square test was applied to detect differences in baseline clinical characteristics between interim PET/CT-positive and PET/CT-negative patients. An elevated LDH level was significantly associated with positive interim PET/CT (*P* < 0.05). The rest of the characteristics were balanced between the two groups.

### Multivariate analysis of prognostic factors associated with OS and PFS

The multivariate analysis showed that interim PET/CT status, age, extranodal involvement, stage, ECOG PS, B symptoms, LDH level and IPI score were not independent prognostic factors associated with PFS or OS (Table 2).

**Table 2:** The multivariate analysis of prognostic factors associated with PFS and OS

	PFS				OS			
	Event / N	3y-PFS% (95%CI)	HR (95%CI)	P	Event / N	3y-OS% (95%CI)	HR (95% CI)	P
Age (years)			0.64 (0.1–19.71)	0.79			0.32 (0.01–199.21)	0.79
≤60	10/103	90.3 (87.4–96.0)			7/103	93.2 (88.3–98.1)		
> 60	2/26	90.8 (84.5–97.1)			0/26	100 (NA)		
Extranodal involvement			1.03 (0.36–2.98)	0.95			0.53 (0.11–2.65)	0.44
No	6/70	91.4 (84.7–98.1)			4/70	94.2 (91.4–97.1)		
Yes	6/59	89.3 (81.3–97.3)			3/70	94.9 (92.1–97.8)		
Ann Arbor Stage			2.81 (0.71–10.41)	0.12			3.87 (0.45–33.5)	0.22
I	2/56	96.0 (93.2–98.8)			1/56	98.1 (96.4–99.8)		
II	10/73	86.3 (78.5–94.1)			6/73	91.8 (88.6–95.0)		
ECOG PS			2.11 (0.36–6.23)	0.18			2.2 (0.49–9.93)	0.31
0	6/73	91.7 (85.4–97.9)			3/73	95.9 (93.6–98.2)		
1	6/56	88.7 (80.1–97.3)			4/56	92.9 (89.5–96.3)		
B symptoms			1.88 (0.36–9.69)	0.45			5.27 (0.81–34.3)	0.08
No	10/120	91.4 (86.3–96.5)			5/120	95.8 (94.1–97.6)		
Present	2/9	77.8 (63.9–91.7)			2/9	77.8 (63.9–91.7)		
LDH level			NA^a^	0.99			NA^a^	0.99
Normal	12/122	89.9 (84.4–96.4)			7/122	94.2 (92.1–96.3)		
Elevated	0/7	100 (NA)			0/7	100 (NA)		
IPI score			NA^a^	0.84			NA^a^	0.82
0–1	12/126	90.3 (85.1–95.6)			7/126	94.4 (92.4–96.4)		
2	0/3	100 (NA)			0/3	100 (NA)		
interim PET/CT			2.4 (0.66–9.03)	0.18			3.68 (0.71–19.12)	0.12
Negative	9/115	91.9 (86.8–97.1)			5/115	95.6 (93.7–97.5)		
Positive	3/14	78.6 (67.6–89.6)			2/14	85.7 (76.3–95.1)		

Abbreviations: *PFS* Progression Free Survival, OS Overall Survival, *LDH* Lactate Dehydrogenase, *IPI* International Prognostic Index, *NA* Not applicable;

* P < 0.05

^a^ Because the sample size is too small, HR can not be calculated

### Comparison of 4R-CHOP+2R and 6R-CHOP

To answer the question of whether the extra 2 cycles of CHOP could be omitted in patients with untreated limited-stage DLBCL without risk factors, a historical control study was performed. Sixty-three patients had negative interim PET/CT scans and no risk factors (primary mediastinal large B-cell lymphoma, extranodal primary or bulky disease) in this study. In NCT 00854568159, there were 128 patients with untreated limited-stage DLBCL without risk factors. There were no significant differences in the baseline demographic and clinical characteristics of the patients between the two groups (Table 3).

**Table 3:** Patients characteristics between two treatment groups

	4R-CHOP+2R current study	6R-CHOP NCT 00854568159	*P* value
Characteristic	No. (%)	No. (%)	
No. of patients	63	128	
Age, year			
Median (range)	47.9 (18–74)	49.3 (18–77)	
≤60y	51 (81)	113 (88.3)	0.25
>60y	12 (19)	15 (11.7)	
Gender			
Male	36 (57.1)	72 (56.2)	0.97
Female	27 (42.9)	56 (43.8)	
Ann Arbor Stage			
I	27 (42.9)	63 (49.2)	0.50
II	36 (57.1)	65 (50.8)	
ECOG PS			
0	40 (63.5)	82 (64)	0.93
1	23 (36.5)	46 (36)	
B symptoms			
No	61 (96.8)	119 (93)	0.46
Yes	2 (3.2)	9 (7)	
LDH level			
Normal	60 (95.2)	120 (93.8)	0.93
Elevated	3 (4.8)	8 (6.3)	
IPI score			
0	51 (81)	106 (82.8)	0.13
1	10 (15.9)	22 (17.2)	
2	2(3.2)	0 (0)	

Abbreviations: *PS* Performance Status, *LDH* Lactate Dehydrogenase, *IPI* International Prognostic Index

The planned dose intensity and drug propensity of the first 4 cycles of R-CHOP were the same in the two groups. The only difference was that patients with negative interim PET/CT scans in this study received another 2 cycles of rituximab monotherapy instead of another 2 cycles of R-CHOP.

**Fig. 3:**
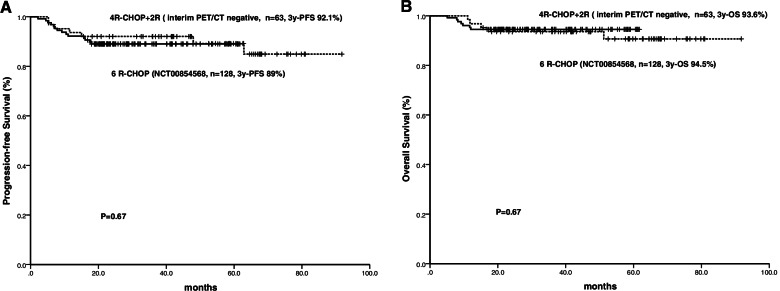
Progression-free survival (**A**) and overall survival (**B**) of patients with 4R-CHOP+2R (interim PET/CT negative) and patients with limited-stage diffuse large B-cell lymphoma treated with 6 R-CHOP in our centre in the same decade (NCT 00854568159)

There were no PFS or OS differences found between patients treated with 4R-CHOP+2R and those treated with 6R-CHOP (3-year PFS 92.1% vs. 89.0%, *P* = 0.67; 3-year OS 93.6% vs. 94.5%, P = 0.67) (Fig. 3).

### Correlation of radiotherapy with survival

Ten patients with negative interim PET/CT underwent radiotherapy after systemic treatment. Six patients with primary testicular lymphoma underwent prophylactic radiotherapy of the contralateral testicle. Two patients with primary paranasal sinus lymphoma, one with primary breast lymphoma, and one with primary thyroid lymphoma received involved field radiotherapy.

There were 43 patients with Waldeyer’s Ring DLBCL in this study. Thirty-nine patients achieved complete response (CR) in interim PET/CT after 4 cycles of R-CHOP and received 2 cycles of rituximab monotherapy, with a 3-year PFS rate of 87.2% and a 3-year OS rate of 89.7%. Involved field radiation therapy was not used in the negative interim PET/CT group.

## Discussion

Recently, the role of interim PET/CT in patients with DLBCL was heavily investigated [21–24]. In this study, patients with negative interim PET/CT showed a good outcome, with a 3-year PFS of 91.9% and a 3-year OS of 95.6%. The survival rates were slightly higher than those reported in previous studies [21–24]. This may be because the patients enrolled in the studies were limited to stage I/II, which was a significant independent prognostic marker for survival in patients with untreated DLBCL.

This was a single-arm study performed to directly answer the question of whether omitting the extra 2 cycles of CHOP would affect efficacy. To minimize the interference of race, region, best of supportive care, etc., we compared the survival data with our historical data of the same period in the same centre. Demographic and clinical characteristics were well balanced between these two cohorts. We found that there were no PFS or OS differences between patients treated with 4R-CHOP+2R and those treated with 6R-CHOP. Reducing the number of chemotherapy cycles has a series of benefits, including avoiding adverse events, cutting economic costs and improving quality of life. Therefore, in the case of similar efficacy, we recommend reducing the number of courses of chemotherapy with the reservation of rituximab. The current study was designed in 2012 as one of earliest studies focusing on interim PET in mainland China. PET-CT scans were not covered by medical insurance. Because the budget for the clinical trial initiated by the investigator was limited and we were not sure about the role of interim PET when we initiated this study ten years ago, we started a preliminary phase 2 clinical trial to observe outcomes according to interim PET guidance. Now, we have confidence to conduct a phase 3, randomized controlled trial based on the current data.

Similarly, to reduce the adverse events caused by excessive chemotherapy, the FLYER trial was conducted to assess the efficacy of de-escalation therapy [11]. In the FLYER trial, they assessed whether 4R-CHOP+2R was noninferior to six cycles of R-CHOP in a population of patients with B-cell non-Hodgkin lymphoma with a favourable prognosis. The 3-year PFS of patients who received 4R-CHOP+2R was 96%, which was 3% better than that of patients who received 6R-CHOP, demonstrating the noninferiority of the 4R-CHOP+2R regimen. Fewer haematological and non-haematological adverse events were documented in the 4R-CHOP+2R group than in the 6R-CHOP group (294 vs. 426, 1036 vs. 1280).

The results of both studies investigating the feasibility of de-escalation therapy suggested that chemotherapy could be reduced without compromising outcomes in some specific DLBCL patients. The enrolled populations of the FLYER study and the current study were not the same. The FLYER study enrolled patients aged 18–60 years with stage I-II disease, normal serum LDH, and ECOG PS 0–1 and without bulky disease (< 7.5 cm). However, patients aged between 18 and 80 years were eligible for the current study. Patients with increased serum LDH were also eligible. Although old age is a poor prognostic factor for DLBCL, the cumulative dose of doxorubicin after 6 cycles reached 300 mg/m^2^. If the subsequent 2 cycles of treatment can be omitted without affecting the efficacy, it will undoubtedly reduce treatment-related toxicity, especially cardiotoxicity. Compared to the FLYER study, we tried to answer the question of whether the number of cycles of CHOP chemotherapy can also be reduced in patients older than 60 years or with increased serum LDH levels. The 3-year PFS of the negative interim PET/CT patients who were older than 60 years or had increased serum LDH levels (*n* = 22) was 89.1% (95% confidence interval 81.8–96.4). The survival data for this subset of patients are similar to the data for all patients. On the other hand, there were four patients who were eligible for the FLYER regimen and had positive interim PET-CT in the current study. These four patients who underwent a total of 8 cycles of R-CHOP had a poor prognosis, with a 3-year PFS of 50% (95% confidence interval 45.1–54.9). Although these patients had such favourable clinical prognostic factors, there may be some molecular genetic poor prognostic factors that were not tested. The sample sizes of those special groups were too small for statistical analysis. However, the results of these two patient groups provide a strong argument for establishing a randomized controlled trial.

The standard treatment of Waldeyer’s ring DLBCL with limited stage remains controversial. Consolidation radiotherapy may prolong survival but can cause many acute or chronic toxicities, such as acute oral mucositis, dental decay, and xerostomia [25, 26]. In the same decade, Li et al. [18] conducted a retrospective study to evaluate the role of consolidation radiotherapy in patients with stage I/II DLBCL limited to Waldeyer’s ring in our centre. The 5-year PFS rates in the immunochemotherapy followed by radiotherapy group vs. the immunochemotherapy alone group were 93.3% vs. 92.5% (*P* = 0.896), and the 5-year OS rates were 96.7% vs. 94.4% (*P* = 0.649). That study found that radiotherapy did not improve the treatment outcomes in patients with limited Waldeyer’s ring DLBCL with CR after R-CHOP therapy. Compared with those results, the PFS and OS in this study were slightly lower. However, 66.7% of the patients in the immunochemotherapy plus radiotherapy group received more than 6 cycles of immunochemotherapy. In contrast, at least 2 cycles of chemotherapy were further omitted in our study. We believe that the results of the 3-year PFS rate of 87.2% and the 3-year OS rate of 89.7% are acceptable as avoiding the adverse events of overtreatment with radiotherapy and chemotherapy.

We tried to find the correlation of interim PET/CT status with baseline demographic and clinical characteristics. An elevated LDH level was significantly associated with positive interim PET/CT (*P* < 0.05). Coinciding with our results, the literature described that elevated LDH levels, bulk and bone marrow involvement and poor performance status lead to more positive interim PET/CT [15]. We failed to find more factors related to interim PET/CT status due to the relatively small sample size of this study. After expanding the sample size, the correlation of interim PET/CT status with characteristics may be more precise.

All factors, including the interim PET/CT status evaluated in the multivariate analysis, are listed in Table 2. No prognostic factors for PFS and OS were found in the current study. Our results contradicted some previous data showing an association between interim PET/CT status and prolonged PFS. However, most of these studies were conducted before the rituximab era [12, 13, 27, 28]. Recently, some studies have eliminated the prognostic value of interim PET/CT and suggested that the use of rituximab could limit the value of interim PET [29, 30]. Another important difference is that in most of the previous studies, interim PET/CT status did not affect the scheduled first-line treatment. However, in the current study, the treatment strategy after 4 cycles of R-CHOP was based on the results of interim PET/CT. Changes in the intensity of subsequent treatment may weaken the prognostic value of the interim PET/CT. Due to the excellent prognosis of early-stage DLBCL patients and our small sample size, we failed to find other characteristic significant independent prognostic markers in the multivariate analysis.

The early results from this preliminary small-scale, phase 2 clinical trial indicated the possibility of downgrading treatment with overall planning for efficacy, safety and economy.

There are some limitations to this study. First, this study is a single-arm, small-scale, phase 2 trial. There is an urgent need for randomized, large-scale trials to answer the question of whether interim PET/CT-negative patients without risk factors could downgrade treatment by omitting 2 cycles of chemotherapy. Second, some new response criteria, such as delta SUV, may be superior for interim PET/CT in DLBCL [31–35]. In future research, we will try to evaluate these new evaluation criteria.

## Conclusion

A trend for inferior outcome was observed in patients with positive interim PET/CT. Our results suggested that for interim PET/CT-negative patients without risk factors, the extra 2 cycles of CHOP might be omitted and radiotherapy might also be omitted in patients with Waldeyer’s ring DLBCL without compromising the efficacy and can avoid the adverse events of overtreatment. These results need to be confirmed in a randomized study.

## Data Availability

The dataset of the current study was available from the corresponding author on reasonable request.

## Abbreviations

DLBCL: Diffuse large B-cell lymphoma
R: Rituximab
CHOP: Cyclophosphamide, doxorubicin, vincristine, prednison
OS: Overall survival
^18^F-FDG: ^18^F-fluoro-2-deoxy-D-glucose
PET: Positron emission tomography
ECOG: Eastern Cooperative Oncology Group
IPI: International Prognostic Index
PS: Performance status
LDH: Lactate dehydrogenase
PR: Partial response
PFS: Progression-free survival
ORR: Objective response rate
NHL: Non-Hodgkin’s lymphoma
CR: Complete response
PD: Progressive disease
